# D-Aspartate Upregulates DAAM1 Protein Levels in the Rat Testis and Induces Its Localization in Spermatogonia Nucleus

**DOI:** 10.3390/biom10050677

**Published:** 2020-04-28

**Authors:** Massimo Venditti, Alessandra Santillo, Sara Falvo, Maria Maddalena Di Fiore, Gabriella Chieffi Baccari, Sergio Minucci

**Affiliations:** 1 Dipartimento di Medicina Sperimentale, Sez. Fisiologia Umana e Funzioni Biologiche Integrate, Università degli Studi della Campania ‘Luigi Vanvitelli’, via Santa Maria di Costantinopoli, 16–80138 Napoli, Italy; massimo.venditti@unicampania.it; 2 Dipartimento di Scienze e Tecnologie Ambientali, Biologiche e Farmaceutiche, Università degli Studi della Campania ‘Luigi Vanvitelli’, Via Vivaldi, 43–81100 Caserta, Italy; alessandra.santillo@unicampania.it (A.S.); sara.falvo@unicampania.it (S.F.); mariamaddalena.difiore@unicampania.it (M.M.D.F.); gabriella.chieffi@unicampania.it (G.C.B.)

**Keywords:** D-Asp, DAAM1, formins, spermatogenesis, cytoskeleton, nuclear actin, mitosis

## Abstract

Cell differentiation during spermatogenesis requires a proper actin dynamic, regulated by several proteins, including formins. Disheveled-Associated-Activator of Morphogenesis1 (DAAM1) belongs to the formins and promotes actin polymerization. Our results showed that oral D-Aspartate (D-Asp) administration, an excitatory amino acid, increased DAAM1 protein levels in germ cells cytoplasm of rat testis. Interestingly, after the treatment, DAAM1 also localized in rat spermatogonia (SPG) and mouse GC-1 cells nuclei. We provided bioinformatic evidence that DAAM1 sequence has two predicted NLS, supporting its nuclear localization. The data also suggested a role of D-Asp in promoting DAAM1 shuttling to the nuclear compartment of those proliferative cells. In addition, the proliferative action induced by D-Asp is confirmed by the increased levels of PCNA, a protein expressed in the nucleus of cells in the S phase and p-H3, a histone crucial for chromatin condensation during mitosis and meiosis. In conclusion, we demonstrated, for the first time, an increased DAAM1 protein levels following D-Asp treatment in rat testis and also its localization in the nucleus of rat SPG and in mouse GC-1 cells. Our results suggest an assumed role for this formin as a regulator of actin dynamics in both cytoplasm and nuclei of the germ cells.

## 1. Introduction

In mammals, spermatogenesis is a highly complex and coordinated process leading to the formation of the mature gametes, the spermatozoa, able to pass through female genital tract and fertilize the oocyte. This progression includes three phases: mitotic proliferation of spermatogonia (SPG), meiotic division of spermatocytes and their differentiation into spermatozoa, the spermiogenesis [1,2]. The whole process is harmonized not only by a cell type- and stage-specific induction or repression of the expression of specific genes [3,4,5,6,7], but it is also regulated by gonadotropins, steroid hormones and a complex network of autocrine and paracrine factors [8,9,10].

Among these, a significant interest in the last decades has been directed to D-aspartate (D-Asp), an endogenous amino acid present in the endocrine and neuroendocrine tissues of vertebrates [11,12,13] particularly in the pituitary gland [11,14] and testes [15,16,17,18,19,20,21]. D-Asp regulates spermatogenesis at two different levels: firstly, acting on the hypothalamus-hypophysis-testis axis, eliciting the release of GnRH [22], the luteinizing hormone (LH) and testosterone [23,24,25,26]; secondly, acting directly on the testis [27,28,29,30]. In fact, D-Asp has also been localized in several germ cells in the rat testis such as in SPG, elongate spermatids [31,32] and spermatozoa [33] other than the interstitial Leydig cells [34]. The role played by D-Asp in steroidogenesis and spermatogenesis is mediated by the activation of several intracellular signaling pathways for review [24]. It is well known that in the Leydig cells the amino acid modulates steroidogenesis by eliciting the adenylate cyclase-cAMP and mitogen-activated protein kinase (MAPK) pathways [11,19,35], while in SPG, D-Asp may directly activate proliferation through both MAPK and AKT pathways [29,35]. However, this current knowledge does not exclude that D-Asp may regulate, activating and/or inhibiting, other molecular pathways.

In this regard, we recently demonstrated that D-Asp increased the expression of the enzyme Prolyl Endopeptidase (PREP) in the rat testis [35], a serine protease having a pivotal role in the regulation of mammalian steroidogenesis and spermatogenesis [36,37,38]. In particular, PREP plays a role in the morphological remodeling, thanks to its co-localization with tubulin [39,40], in the cytoplasm of Sertoli and germ cells, as well as in the nucleus of SPG and spermatocytes, suggesting its role in the proliferative phases of spermatogenesis [35].

As cytoskeleton remodeling is an integral aspect of spermatogenesis, and thus essential for male fertility, an understanding of the multitude of proteins and factors that regulate their dynamics, is of crucial importance [41]. In our previous work [42] we characterized one of these proteins, Disheveled-Associated Activator of Morphogenesis 1 (DAAM1) during the post-natal development of the rat testis, and in rat and human sperm, implying its possible role in reproduction. DAAM1 is a multidomain protein belonging to the formins, a large family that play key roles in the regulation of actin polymerization [43].

Thus, to better understand the underlying molecular pathways responsible for the action of D-Asp in the rat testis, here, we examined the effects of oral administration of D-Asp on DAAM1 protein levels and localization in adult rats.

## 2. Materials and Methods

### 2.1. Animals and Experiments

Male Wistar rats (*Rattus norvegicus*), weighing 300 to 350 g, were kept under regulated temperature (24 ± 2 °C) and lighting conditions (12 h light and 12 h dark cycles). They received commercial food pellets *ad libitum*. Rats (*n* = 10) were divided into two groups: the first group (*n* = 5) was allowed to drink a solution consisting of 20 mM D-aspartate (D-Asp; 219096; Sigma-Aldrich, Milan, Italy) for 15 days [18,35]; rats in the second group (control, *n* = 5) were given fresh water for 15 days. At the end of the treatment, in the early daylight hours, rats were first anesthetized by an intraperitoneal injection of chloral hydrate (47335-U; Sigma-Aldrich, Milan, Italy) and then sacrificed. The testes were dissected out, weighed and rapidly immersed in Bouin’s fluid (solution of picric acid, glacial acetic acid and formaldehyde; 05-01008Q; Bio-Optica, Milan, Italy) and liquid nitrogen (SOL; Caserta, Italy) for histochemical and biochemical analyses, respectively [44]. The experimental protocol and the housing conditions were in accordance with the Italian guidelines (D. Lvo 116/92) and authorized by the local Animal Care Committee (Servizio veterinario ASL 44, Prot. Vet. 22/95).

### 2.2. Cell Culture and Treatments

A cell line derived from immortalized type-B mouse spermatogonia (SPG) retaining markers of mitotic germ (GC-1 SPG; CRL-2053; ATCC, Manassas, VA, USA) were cultured in Dulbecco’s modified Eagle’s Medium (D-MEM; D6429; Sigma-Aldrich, Milan, Italy), supplemented with 10% fetal bovine serum (FBS) (10082147; Gibco BRL, Milan, Italy) and grown in a 37 °C humidified atmosphere of 5% CO_2_ [45]. Cultures were carried out in the presence of 200 µM D-Asp for 30 min [29,35]. Control cultures were incubated for the same times with vehicle alone. At the end of the incubation period, cells were rinsed in phosphate-buffered saline (PBS) (D8537; Sigma-Aldrich, Milan, Italy), harvested by trypsinization (T4174; Sigma-Aldrich, Milan, Italy), collected by centrifugation at 1000 g for 10 min and immediately frozen at −80 °C for biochemical analyses or fixed for 20 min with a 4% (*w*/*v*) paraformaldehyde solution (158127; Sigma-Aldrich, Milan, Italy) and spotted on a slide for immunofluorescence study.

### 2.3. Preparation of Total Protein Extracts from Rat Testis and GC-1 Cells and Western Blotting Analysis

Rat testes and cell pellets were lysed in lysis buffer (all reagents from Sigma-Aldrich, Milan, Italy) containing 50 mM Tris HCl (pH 7.5), 5 mM EDTA, 300 mM NaCl, 150 mM KCl, 1 mM dithiothreitol, 1% Nonidet P40 in PBS (13.6 mMNaCl; 2.68 mMKCl; 8.08 mM Na_2_HPO4; 18.4 mM KH_2_PO4; 0.9 mM CaCl_2_; 0.5 mM MgCl_2_; pH 7.4) and a mix of protease inhibitors (P8340; Sigma-Aldrich, Milan, Italy) [4 μg/μL of leupeptin, aprotinin, pepstatin A, chymostatin, phenylmethylsulfonyl fluoride (PMSF) and 5 μg/μL of tosyl phenylalanyl chloromethyl ketone (TPCK)]. The protein extracts (30 µg) were boiled in Laemmli sample buffer (#1610747; BioRad; Melville, NY, USA), separated using a 12% sodium dodecyl sulfate-polyacrylamide gel electrophoresis (SDS–PAGE) and transferred onto a nitrocellulose membrane (#1620115; BioRad; Melville, NY, USA). The complete transfer was assessed using a pre-stained protein marker (#1610373; BioRad; Melville, NY, USA). Western blotting was repeated three times. Membranes were first treated for 1 h with a blocking solution (5% non-fat powdered milk in 25 mM Tris, pH 7.4; 200 mM NaCl; 0.5% Triton X–100, TBS/Tween) (all reagents from Sigma-Aldrich, Milan, Italy) and then incubated overnight at 4 °C with the primary antibody: (1) anti-DAAM1 diluted 1:1000 (SAB2100527; Sigma-Aldrich, Milan, Italy); (2) anti-PCNA diluted 1:1000 (#98825; Sigma-Aldrich, Milan, Italy); (3) anti-p-H3 diluted 1:1000 (#06-570; Merck Millipore, Darmstadt, Germania); and (4) anti-β-actin diluted 1:1000 (bs-0061R; Bioss Antibodies, Woburn, Massachusetts, USA). After washing thrice with TBS/Tween, the membranes were incubated with horseradish peroxidase-conjugated secondary antibody (12–348; Sigma-Aldrich; Milan, Italy) diluted 1:5000 in the blocking solution for 1 h at room temperature. Then, membranes were washed again thrice in TBS/Tween and the immunocomplexes were detected using an enhanced chemiluminescence (ECL) system (RPN2106; Amersham Bioscience, Buckinghamshire, UK) [46]. Protein bands were then quantified using the ImageJ, a free software for digital image processing, based on Sun-Java, developed by the National Institutes of Health of the United States. The band from each sample was quantified three times. The amount of the proteins was normalized with respect to β-actin protein.

### 2.4. Preparation of Nuclear Protein Extracts from Rat Testis and Western Blotting Analysis

For the analysis on nuclear and cytosolic proteins, adult testes (*n* = 5 from each group) were gently homogenized using a type B pestle, in seven volumes (*w*/*v*) of hypotonic Hepes buffer (10 mM Hepes, pH 7.9, 1.5 mM MgCl2, 10 mM KCl, 12% glycerol, 0.1 mM EGTA, 0.5 mM dithiothreitol, 0.5 mM spermidine) (all reagents from Sigma-Aldrich, Milan, Italy) with protease inhibitors (P8340; Sigma-Aldrich, Milan, Italy; 4 μg/mL of leupeptin, aprotinin, pepstatin A, chymostatin, PMSF and 5 μg/mL of TPCK). After centrifugation at 800 g, the supernatant was removed and nuclear pellet, washed three times, resuspended in 1.2 volumes (1.2 mL/mg pellet) of hypertonic Hepes buffer (10 mM Hepes, pH 7.9, 1.5 mM MgCl2, 420 mM NaCl, 15% glycerol, 0.1 mM EGTA, 0.5 mM dithiothreitol, 2 mM spermidine) (all reagents from Sigma-Aldrich, Milan, Italy) with protease inhibitors (see above) and stirred at 4 °C for 30 min. Nuclei were pelleted by centrifugation at 10,000 g for 30 min at 4 °C. The supernatants, containing nuclear proteins were collected [37]. Cytosolic and nuclear extracts (50 µg) were boiled in Laemmli sample buffer (#1610747; BioRad; Melville, NY, USA), separated using a 12% SDS–PAGE and transferred onto a nitrocellulose membrane (#1620115; BioRad; Melville, NY, USA). The complete transfer was assessed using a pre-stained protein marker (#1610373; BioRad; Melville, NY, USA). Western blotting was repeated three time. Membranes were first treated for 1 h with a blocking solution (5% non-fat powdered milk in 25 mM Tris, pH 7.4; 200 mM NaCl; 0.5% Triton X–100, TBS/Tween) (all reagents from Sigma-Aldrich, Milan, Italy) and then incubated overnight at 4 °C with the primary antibody: (1) anti-DAAM1 diluted 1:1000 (SAB2100527; Sigma-Aldrich, Milan, Italy); (2) anti-H3 diluted 1:2000 (#06-755; Merck Millipore, Darmstadt, Germania); and (3) anti-β-actin diluted 1:1000 (bs-0061R; Bioss Antibodies Woburn, Massachusetts, USA). After washing thrice with TBS/Tween, the membranes were incubated with horseradish peroxidase-conjugated secondary antibody (12-348; Sigma-Aldrich; Milan, Italy) diluted 1:5000 in the blocking solution for 1 h at room temperature. Then, membranes were washed again thrice in TBS/Tween and the immunocomplexes were detected using an ECL system (RPN2106; Amersham Bioscience, Buckinghamshire, UK). Protein bands were then quantified using the ImageJ. The band from each sample was quantified three times. The amount of the proteins was normalized with respect to the β-actin protein.

### 2.5. Immunofluorescence Analysis on Rat Testis

For DAAM1 co-localization with actin, 5 µm-testis sections were dewaxed, rehydrated and processed as described by Chemek et al. [47]. Antigen retrieval was performed by pressure cooking slides for 3 min in 0.01 M citrate buffer (pH 6.0). Then, the slides were incubated with 0.1% (*v*/*v*) Triton X-100 in PBS for 30 min. Later, nonspecific binding sites were blocked with normal goat serum diluted 1:5 in PBS containing 5% (*w*/*v*) bovine serum albumin (BSA; A2153; Sigma–Aldrich, Milan, Italy) before the addition of anti-DAAM1 and anti- β-actin antibody diluted 1:100, for overnight incubation at 4 °C. After washing in PBS, slides were incubated for 1 h with the appropriate secondary antibody (#A-11008; Alexa Fluor 488, Invitrogen; FITC Jackson, ImmunoResearch, Pero MI, Italy; SAB4600082; Anti-Mouse IgG 568, Sigma–Aldrich, Milan, Italy) diluted 1:500 in the blocking mixture. The slides were mounted with Vectashield + DAPI (4′,6-diamidino-2-phenylindole; H-1200-10; Vector Laboratories, Peterborought, UK) for nuclear staining and then observed under an optical microscope (Leica DM 5000 B + CTR 5000). Images where viewed and saved with IM 1000 software. Densitometric analysis of DAAM1 immunofluorescence was performed with ImageJ Software counting 30 seminiferous tubules/animal (five controls and five D-Asp treated rats) for a total of 150 tubules per group.

### 2.6. Immunofluorescence Analysis on GC-1

Cells were permeabilized for 10 min with 0.1% (*w*/*v*) Triton X-100 in PBS at room temperature. Later, nonspecific binding sites were blocked with normal goat serum diluted 1:5 in PBS containing 5% (*w*/*v*) BSA before the addition of anti-DAAM1 and anti- β-actin antibody diluted 1:100, for overnight incubation at 4 °C. After washing in PBS, slides were incubated for 1 h with the appropriate secondary antibody (#A-11008; Alexa Fluor 488, Invitrogen; FITC Jackson, ImmunoResearch, Pero MI, Italy; SAB4600082; Anti-Mouse IgG 568, Sigma–Aldrich, Milan, Italy) diluted 1:500 in the blocking mixture. The slides were mounted with Vectashield + DAPI (H-1200-10; Vector Laboratories, Peterborought, UK) for nuclear staining and then observed under the optical microscope (Leica DM 5000 B + CTR 5000) and images where viewed and saved with IM 1000 software. We performed two different negative controls: (1) by omitting the primary antibody and (2) by using rat isotype IgG (#I5006, Sigma-Aldrich, Milan, Italy) Densitometric analysis of DAAM1 immunofluorescence was performed with ImageJ Software. Fifty cells, either control and D-Asp treated GC-1, were counted in three different immunofluorescent experiments, for a total of 150 each. Moreover, DAAM1 colocalization analysis with β-actin or nucleus was performed using the Intensity correlation analysis plug-in incorporated into ImageJ.

### 2.7. Statistical Analysis

The values obtained were compared by Student’s *t*-test for between-group comparisons. The differences were considered statistically significant at *p* < 0.05. All data were expressed as the mean ± standard deviation (SD).

## 3. Results

### 3.1. Effects of Oral Administration of D-Asp on Testicular DAAM1 Protein Levels and Localization

**Figure 1:**
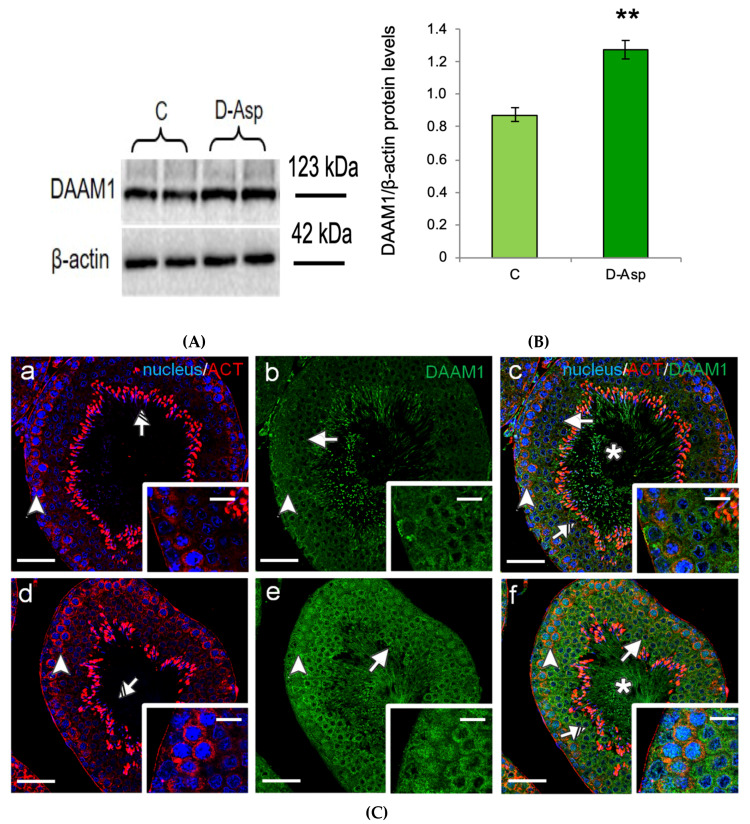

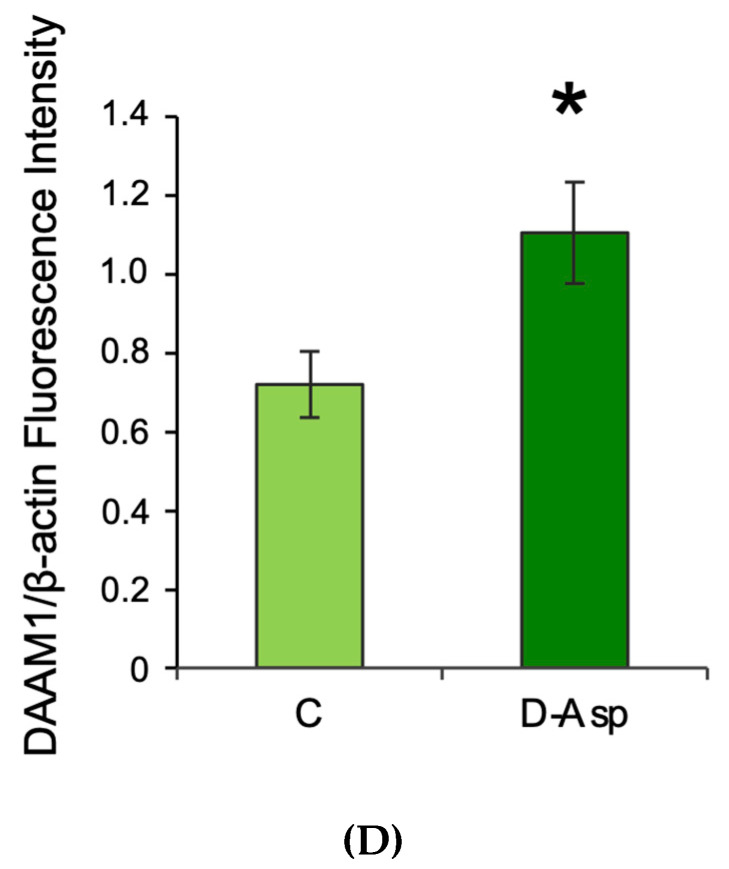
Testicular Disheveled-Associated-Activator of Morphogenesis1 (DAAM1) protein levels and localization in D-Asp treated rats. (**A**) Western blot analysis of DAAM1 (123 kDa) protein levels in the testis from D-Asp-treated and control rats. (**B**) The amount of DAAM1 was quantified using ImageJ program and normalized with respect to β-actin (42 kDa). Values represent the means ± S.D. of five samples. ** *p* < 0.01 *versus* controls. (**C**) DAAM1 and actin co-localization in the testis from controls (**a**–**c**) and D-Asp-treated rats (**d**–**f**). (**a**,**d**) DAPI-fluorescent nuclear staining (blue) and β-actin (ACT) localization (red). (**b**,**e**) DAAM1 fluorescence (green). (**c**,**f**) Merged fluorescent channels (blue/red/green). The intermediate yellow-orange and light-blue tints reflect DAAM1 co-localization with β-actin within the cytoplasm and in the nucleus, respectively. The images in the insets were captured at ×40 magnification, all the others at ×20 magnification. Scale bars represent 20 μm, except for the insets, where they represent 10 μm. Arrowheads: Spermatogonia; Arrows: Spermatocytes. Striped Arrows: Spermatids; Asterisks: luminal Spermatozoa. (**D**) Histogram showing the quantification of DAAM1 fluorescence signal intensity whit respect to β-actin signal using ImageJ. Values represent the means ± S.D. of three separate experiments. * *p* < 0.05 *versus* controls.

The oral administration of D-Asp induced a significant increase in the testicular protein levels of DAAM1 as compared to those of control rats (*p* < 0.01; Figure 1A,B). In order to characterize the modulation of DAAM1 protein levels by D-Asp, a double-immunofluorescence with its cytoskeletal partner, actin, was performed on rat testis (Figure 1C).

In particular, in the control rats (Figure 1C (a–c)), we observed that DAAM1 was mainly localized in the cytoplasm of mitotic and meiotic cells (arrows, Figure 1C (b,c) and insets), showing a more intense signal in the latter, as well as in the cytoplasm of spermatids and in the luminal spermatozoa (asterisk, Figure 1C (c) and inset). Moreover, the results revealed a co-localization of DAAM1 with β-actin in the perinuclear cytoplasm of spermatogonia (SPG), highlighted by the intermediate yellow-orange tint (arrowhead, Figure 1C (c) and inset), as well as a very strong signal for β-actin in maturing late spermatids (striped arrow, Figure 1C (a,c) and inset). A positive staining was also found in the somatic cells, particularly in Sertoli, Leydig and peritubular cells. In D-Asp treated rat testis (Figure 1C (d–f)) we found that DAAM1 immunofluorescent signal was more intense in the cytoplasm of all the cells composing the seminiferous epithelium compared to the controls (Figure 1C (e,f) and insets). Surprisingly, a clear nuclear signal for DAAM1 appeared in type B SPG, underlined by the light-blue staining (arrowhead, Figure 1C (e,f) and insets). It has to be highlighted that the perinuclear co-localization of DAAM1 with β-actin is more pronounced in these cell types (arrowhead, Figure 1C (f) and inset). DAAM1 immunofluorescent signal following the oral administration of D-Asp showed a significant increase as compared to those of control rats (*p* < 0.05 Figure 1D). Finally, the specificity of DAAM1 antisera was tested performing two different negative controls: by omitting the primary antibody and using rat isotype IgG, without giving any fluorescent signal (data not shown) [35].

### 3.2. DAAM1 Nuclear Localization: Bioinformatics and Biochemical Analysis

**Figure 2:**
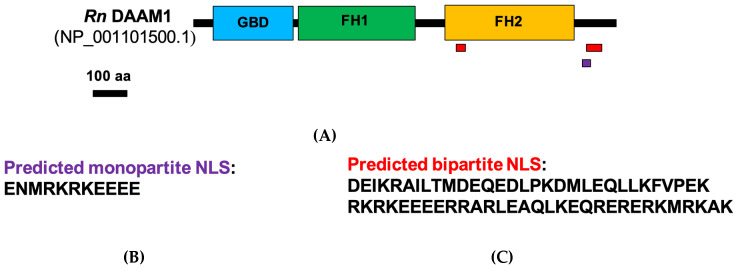
Schematic representation of domain architecture of rat DAAM1. (**A**) The main domains are represented: GTPase binding domain (GBD) domain (blue), Formin Homology 1 (FH1) domain (green) and Formin Homology 2 (FH2) domain (yellow). (**B**) Amino acid sequence of the predicted monopartite nuclear localization signal (NLS) (purple). (**C**) Amino acid sequence of the predicted bipartite NLS (red).

To assess the possible nuclear localization of rat DAAM1 via a bioinformatics approach, we investigated its sequence to identify the possible presence of a nuclear localization signal (NLS). In Figure 2A, a diagram of domain architecture of rat DAAM1 is represented, showing the three major domains of a typical formin: the GTPase binding domain (GBD, in blue), the Formin Homology 1 domain (FH1, green) and Formin Homology domain 2 (FH2, yellow) [48].

By the use of the free software NLS mapper (http://nls-mapper.iab.keio.ac.jp/cgi-bin/NLS_Mapper_form.cgi), we evidenced two predicted NLS in a DAAM1 sequence. Both signals are localized at the C-terminal of the protein: a monopartite NLS (purple) and a bipartite NLS (red; Figure 2B,C) [49].

**Figure 3:**
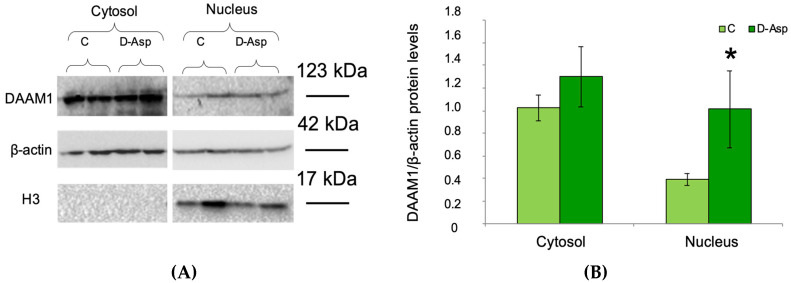
DAAM1 (123 kDa) levels in cytosolic and nuclear protein fractions in D-Asp treated rats. (**A**) Western blot detection of DAAM1 protein in cytosolic and nuclear fractions of D-Asp-treated and control rat testis. To evaluate the purity of the samples, the nuclear protein levels of histone H3 (17 kDa) was detected. (**B**) The amount of DAAM1 was quantified using ImageJ program and normalized with respect to β-actin (42 kDa). Values represent the means ± S.D. of five samples. * *p* < 0.05 *versus* controls.

Having verified that DAAM1 possesses the predicted NLS sequence that could allow its shuttling in the cell nucleus, we proceeded with a Western blotting performed on separated cytosolic and nuclear fractions. This analysis provided evidences of the effective expression of DAAM1 in the two extract fractions (Figure 3A,B). Furthermore, D-Asp administration induced an increase in DAAM1 protein levels in both cytosolic and nuclear fractions (*p* < 0.05 for nuclear fraction; Figure 3A,B).

### 3.3. Effects of D-Asp on DAAM1 Protein Levels and Localization in Mouse GC-1 Cells

**Figure 4:**
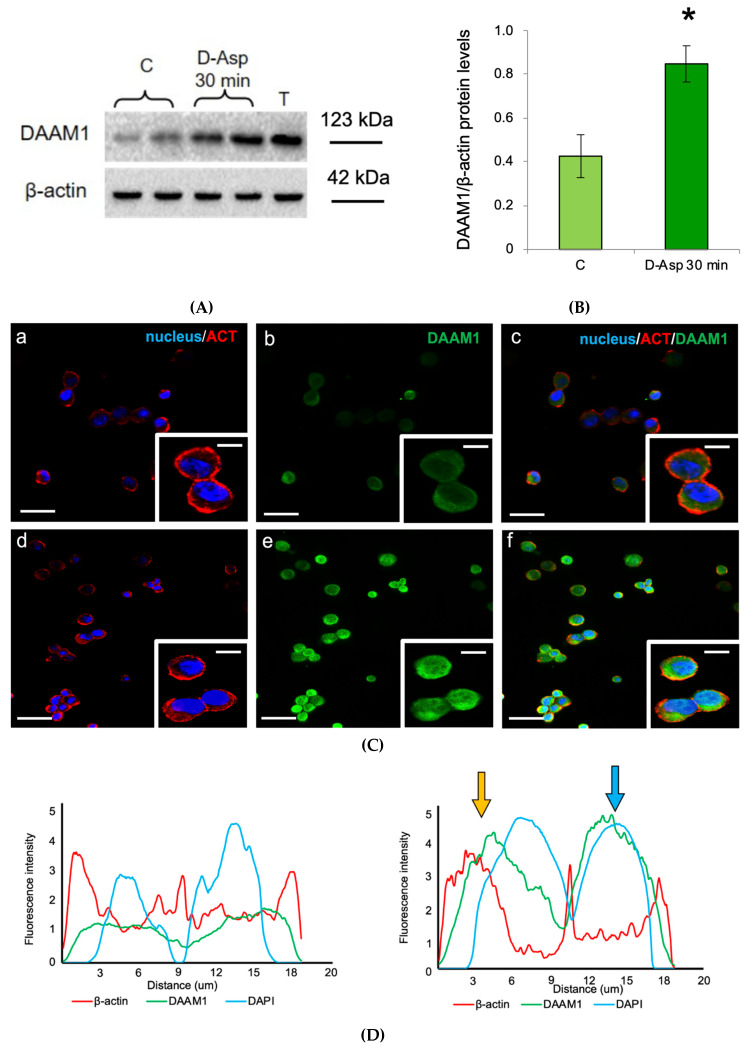

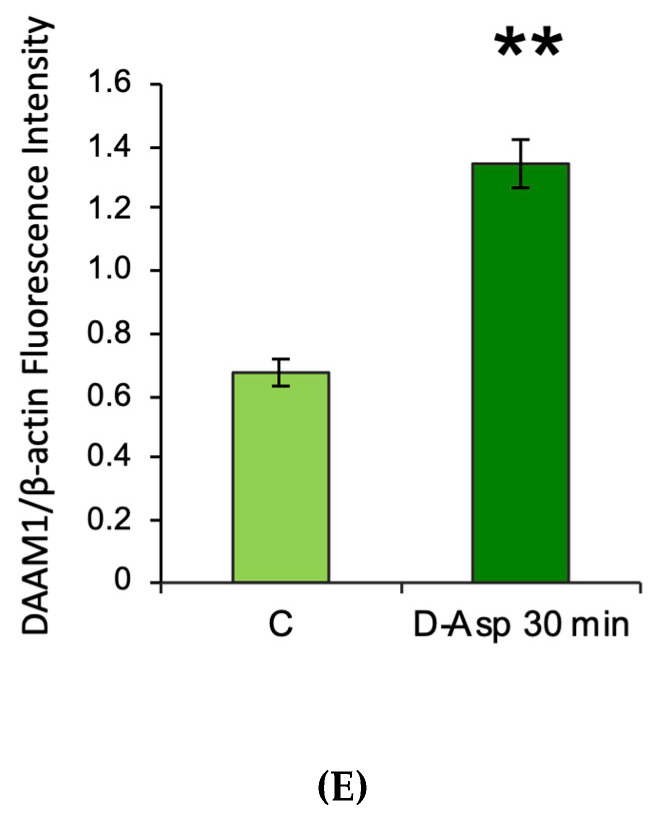
DAAM1 protein levels and localization in cultured mouse GC-1 cells. (**A**) DAAM1 Western blot detection (123 kDa) in GC-1 cells at 30 min after D-Asp treatment and at time 0 (control, C); T = Testis. (**B**) DAAM1 protein levels are quantified using the ImageJ program and normalized with respect to β-actin (42 kDa). Values represent the means ± S.D. of three separate experiments. * *p* < 0.05 *versus* controls. (**C**) DAAM1 and β-actin co-localization in controls (**a**–**c**) and insets) and in D-Asp treated mouse GC-1 cultured cells (**d**–**f**) and insets). (**a**,**d**) DAPI-fluorescent nuclear staining (blue) and β-actin (ACT) localization (red). (**b**,**e**) DAAM1 fluorescence (green). (**c**,**f**) merged fluorescent channels (blue/red/green). The intermediate yellow-orange and light-blue tints reflect DAAM1 co-localization with β-actin and in the nucleus, respectively. The images of the magnifications were captured at ×40 magnification, all the others at ×20 magnification. Scale bars represent 20 μm, except for the magnifications, where they represent 10 μm. (**D**) Plot profiles of normalized pixel intensity of DAAM1 (green), β-actin (red) and DAPI (blue) corresponding to the GC-1 cells highlighted in the insets. Analysis of colocalization of β-actin (red curves) and DAPI (blue curves) with DAAM1 (green curve) in control (on the left) and D-Asp treated GC-1 cells. The fluorescence intensity profiles show the distribution of fluorescence across the dotted line (*x*-axis). The fluorescence intensities are plotted along the *y*-axis. Arrows indicate DAAM1-β-actin (yellow) and DAAM1-DAPI (light blue) co-localization. (**E**) Histogram showing the quantification of DAAM1 fluorescence signal intensity with respect to β-actin using ImageJ. Values represent the means ± S.D. of three separate experiments. ** *p* < 0.01 *versus* controls.

Since the immunofluorescence analysis in the rat testis revealed a specific DAAM1 nuclear localization in SPG, we extended the analysis on mouse cultured GC-1 cells [29,30,50,51]. We found that the addition of D-Asp to the culture medium induced a significant increase in DAAM1 protein levels at 30 min with respect to control (*p* < 0.05; Figure 4A,B).

The immunofluorescence analysis confirmed this trend: DAAM1 and actin are co-localized in the cytoplasm of non-treated GC-1 cells, close to the nuclear region (Figure 4C (a–c) and insets); however, following D-Asp treatment, their signal intensity increased, as can be seen by the intermediate yellow-orange tint given by the merge of the images. Moreover, the DAAM1 signal appeared inside the nucleus, where the light-blue staining reflects this sub-localization, corroborating the results obtained in the rat testis (Figure 4C (d–f) insets). As a support of the above data, the plot profiles showed in Figure 4D help to validate DAAM1 nuclear localization: each line represent the spatial distribution of the fluorescent intensity; the matching of the peaks indicates a putative co-localization of the two signals. On the left is the profile of the control cells and on the right that of D-Asp treated GC-1, in the latter the arrows point to DAAM1-β-actin (yellow) and DAAM1-DAPI (light-blue) co-localization. It is interesting to note that DAAM1-β-actin co-localization is particularly pronounced just “outside” the blue peak, in correspondence to the perinuclear region. Finally, the increased DAAM1 immunofluorescent signal following the oral administration of D-Asp as compared to those of control rats is shown in Figure 4E (*p* < 0.01).

### 3.4. Effects of Oral Administration of D-Asp on Testicular PCNA and p-H3 Protein Levels

**Figure 5:**
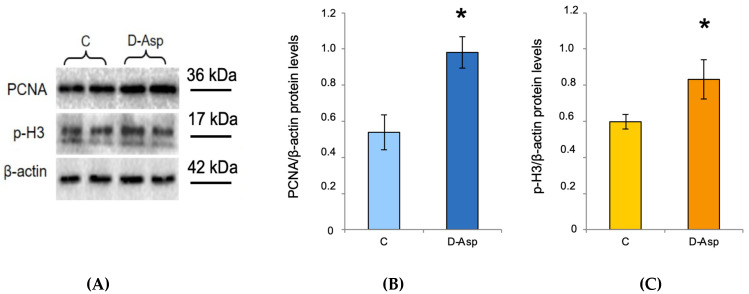
Testicular PCNA and p-H3 protein levels in D-Asp treated rats. (**A**) Western blot analysis of PCNA (36 kDa) and p-H3 (17 kDa) proteins in the testis from D-Asp-treated rats and controls. (**B**) The amount of PCNA was quantified using ImageJ program and normalized with respect to β-actin (42 kDa). Values represent the means ± S.D. of five samples. * *p* < 0.05 *versus* controls. (**C**) The amount of p-H3 was quantified using the ImageJ program and normalized with respect to β-actin. Values represent the means ± S.D. of five samples. * *p* < 0.05 *versus* controls.

PCNA, a protein expressed in the nucleus of cells in the S phase [52] and p-H3, a histone crucial for chromatin condensation during mitosis and meiosis [53], were used as markers of cell cycle progression. PCNA and p-H3 levels were significantly higher in the testis of rats treated with D-Asp as compared to the those observed in the testis of control rats (Figure 5A–C).

## 4. Discussion

All the eukaryotic cells are characterized by the presence, in their cytoplasm, of an intricate network of filamentous elements which represent the cytoskeleton, which is composed of: actin microfilaments, intermediate filaments and microtubules. Each of them is essential to cell life because they participate in many functions, including intracellular trafficking, division, migration, motility, adhesion, differentiation and cell shape determination see for review [54].

In particular, the microfilaments are formed by the non-covalent assembly of globular actin monomers into double helical filaments (F-actin) [55]. Actin polymerization usually occurs through two systems, namely, the Arp2/3 complex and the formins [56]. As a large group of proteins, formins share a highly conserved domain or the Formin Homology 2 (FH2) domain, responsible of the F-actin assembly [57], while the flanking regions that are involved in numerous cellular functions and regulatory mechanisms vary considerably [58].

A component of this protein family, Disheveled-Associated Activator of Morphogenesis 1 (DAAM1) can control the nucleation and assembly of actin fibers in response to several signals [43]. In our previous work, we demonstrated that DAAM1 might be directly involved in the dramatic cytoskeletal-dependent events that occur during the post-natal development of male rat gonads [42]. Indeed, male gametogenesis is accurately controlled by the interplay of many endocrine, paracrine and autocrine factors [59]. Beyond the classical modulators of the hypothalamus-pituitary-gonad (HPG) axis, the D-Aspartate (D-Asp) is one of those that attracted great interest. Indeed, D-Asp, being an excitatory amino acid, regulates the spermatogenesis eliciting the hormone release by the HPG axis and the spermatogonia (SPG) proliferation via the activation of several intracellular signaling pathways [25,29,60]. With the aim to expand upon the current knowledge concerning other possible molecules whose expression could be modulate by D-Asp, we focused our attention only on the formin DAAM1, also considering that recently we showed that this amino acid increased the expression of a microtubule-associated protein, the serine protease Prolyl Endopeptidase, in the rat testis [35].

The results obtained from chronic oral administration of D-Asp to adult rats for 15 days revealed, for the first time, that the testicular DAAM1 protein levels increased. Furthermore, the immunofluorescence analysis showed a stronger immunopositivity in the cytoplasm of all the germ cells types of the exposed rats as compared to the control. One of the crucial aspects of gamete production is their differentiation and intimate changes in shape and morphology, a cytoskeleton-dependent process [41]. This suggests a possible role for D-Asp in the regulation of DAAM1 protein levels during germ cells differentiation, when an intensive actin state modification and cytoskeletal rearrangement occurs [61]. This hypothesis is also supported by the co-localization of DAAM1 and actin in the testicular cells of both control and D-Asp treated rats.

Worthy of note is that this formin is activated by WNT ligands, in order to either recruit actin at the FH2 domain or interact with the small GTPase RhoA [62], which activates Rho-associated protein kinase (ROCK) [63], provoking the actin and cytoskeletal remodeling [64].

Interestingly, DAAM1 and RhoA play a key role in several events, such as gastrulation [62] and synaptic plasticity [65,66]. Particularly, in the excitatory neurons, the GTPase can be activated by various signals, including WNT [67] and the stimulation of glutamate receptors, as NMDARs and mGluRs [68]. Although D-Asp specific receptors have not been identified yet, a number of reports indicate that NMDAR and AMPAR have affinity for it [30,69]. Of note is that such receptors have been detected in rodent testis and their expression increased following D-Asp administration [18,30]. Therefore, we hypothesize that in the rat testis, DAAM1 might be a possible key player in the AMPAR-dependent activation of molecular pathway elicited by D-Asp, thus regulating the massive actin remodeling that occurs during germ cells proliferation and differentiation.

Noteworthy, this work provided the first evidence for the nuclear localization of DAAM1. Immunofluorescence analysis revealed the presence of DAAM1 specifically in type B SPG nuclei, where the perinuclear co-localization of DAAM1 with actin is more pronounced. In addition, this result has been confirmed by a Western blotting analysis performed on the nuclear and cytoplasmic extracts of the D-Asp treated and control whole testis, in which not only the nuclear presence of DAAM1 is demonstrated, but also its increase following the exposure. To our knowledge, it has never been reported in the literature that this formin, believed to be exclusively a cytoplasmic protein, may also be localized in the cell nucleus. Thus, our data suggest that DAAM1 might act in the actin polymerization mechanisms within the nucleus.

In the 1970s, many studies reported the presence of actin and proteins related to actin in the nucleus [70]. This discovery caused a lot of skepticism in the scientific community for a long time. Indeed, although actin is one of the most abundant protein in eukaryotic cytoplasm, its amount in the nucleus is relatively low, leading to the difficulty to highlight monomeric or polymeric actin by conventional techniques [71,72,73]. However, over the years, several studies have been published reporting that the nuclear pool of actin exists in many forms and that its dynamics are finely regulated, suggesting that it plays important roles in the nucleus [74]. In fact, nuclear actin has been widely implicated in transcription regulation, chromatin remodeling, DNA-damage repair, and DNA replication see for review [70].

Low levels of nuclear actin are regulated by either active transport of monomers between the cytoplasm and nucleus [75,76] and its local polymerization [77]. This balance implies the nuclear presence of other proteins controlling the whole process; for instance, cofilin and profilin allow the import by Importin-9 [76] and export by Exportin-6 of actin in the nucleus, respectively [75]. Despite that formins have been historically associated with the cytoplasmic actin dynamics [55], several studies demonstrated that some of them can be shuttled in and out the nucleus [78], since different formins have been shown to have functional nuclear localization signal (NLS) [79,80,81,82]. For example, in apoptotic cells, the C-terminal tail of the formin FHOD1 is cleaved by caspase-3 and, consequently, targeted to the nucleus [83], while mDia2, another formin, may stimulate serum- responsive factor-dependent gene transcription by promoting F-actin assembly in the nucleus [84]. In this paper, we provided a bioinformatic evidence that DAAM1 sequence has two predicted NLS (a monopartite and a bipartite NLS), strengthening the hypothesis that, in specific conditions, it could be shuttled to the nucleus and participate in the actin polymerization. However, the precise molecular mechanism by which DAAM1 could be shuttled within the nucleus and its interaction with the pool of nuclear actin are still unknown and further studies will need to address this. Furthermore, since we observed an increase of the nuclear DAAM1 protein levels particularly in type B SPG, in order to confirm this result, we conducted an in vitro experiment on mouse SPG cultured cells (GC-1), which showed characteristics of a stage between type B SPG and primary spermatocytes. In fact, in these cells, following the exposure, the protein levels of the formin significantly increased and with immunofluorescent analysis, we evidenced the appearance of DAAM1 signal in the nucleus. Altogether, this crucial aspect has been confirmed through different methods: bioinformatic study (evidencing two predicted NLS), Western blotting (demonstrating DAAM1 nuclear presence) and immunofluorescence staining and relative image analysis (showing DAAM1 co-localization with DAPI, increased following D-Asp treatment). In addition, considering that SPG are the proliferative cells, which, undergoing different mitosis cycles, allow the self-renovation of seminiferous cells, here, we analyzed the expression of PCNA [52] and p-H3 [53] to verify the proliferative action induced by D-Asp. In our previous study, we demonstrate that D-Asp induced proliferation in spermatogonia GC-1 cells [29], stimulating expression of PCNA and Aurora B via ERK and Akt pathways. Activation of ERK1/2 pathway by D-Asp has also been observed in testis from D-Asp-treated adult rats [18]. Consistently, we found an increase in PCNA and P-H3 protein levels in D-Asp-treated testes. Thus, we hypothesize that DAAM1 shuttling in the nucleus following D-Asp treatment could be required as a response to the demand of the enhanced proliferation. Indeed, actin and its dynamic regulated by formins, are necessary to control cell proliferation by multiple mechanisms [85], including DNA replication. On the other hand, Parisis et al. have demonstrated that formin inhibition abolishes nuclear transport of actin, which is essential for DNA replication in proliferating cells [86]. Thus, we can suggest a double action exerted by DAAM1 in the germ cells: in the cytoplasm, by regulating actin remodeling in the differentiation phases of the seminiferous cycle, and in the nucleus of type B SPG, to allow actin polymerization, a crucial event for DNA replication and eventually cell division.

In conclusion, herein we have demonstrated for the first time an increased DAAM1 protein levels following D-Asp treatment in rat testis. Interestingly, after the treatment, DAAM1 was also localized in the nucleus of rat type B SPG and in mouse GC-1 cells. These results support a pivotal role of this protein in the regulatory processes promoting actin dynamics in both cytoplasm and nucleus of germ cells, for their differentiation. Further studies are needed to confirm this suggestive hypothesis.
